# VTG-Net: A CNN Based Vessel Topology Graph Network for Retinal Artery/Vein Classification

**DOI:** 10.3389/fmed.2021.750396

**Published:** 2021-11-08

**Authors:** Suraj Mishra, Ya Xing Wang, Chuan Chuan Wei, Danny Z. Chen, X. Sharon Hu

**Affiliations:** ^1^Department of Computer Science and Engineering, University of Notre Dame, Notre Dame, IN, United States; ^2^Beijing Ophthalmology and Visual Sciences Key Laboratory, Beijing Institute of Ophthalmology, Beijing Tongren Hospital, Capital Medical University, Beijing, China; ^3^Department of Ophthalmology, Beijing Tongren Hospital, Capital Medical University, Beijing, China

**Keywords:** retinal images, artery/vein classification, vessel topology, convolutional neural networks, graph convolutional networks

## Abstract

From diagnosing cardiovascular diseases to analyzing the progression of diabetic retinopathy, accurate retinal artery/vein (A/V) classification is critical. Promising approaches for A/V classification, ranging from conventional graph based methods to recent convolutional neural network (CNN) based models, have been known. However, the inability of traditional graph based methods to utilize deep hierarchical features extracted by CNNs and the limitations of current CNN based methods to incorporate vessel topology information hinder their effectiveness. In this paper, we propose a new CNN based framework, VTG-Net (vessel topology graph network), for retinal A/V classification by incorporating vessel topology information. VTG-Net exploits retinal vessel topology along with CNN features to improve A/V classification accuracy. Specifically, we transform vessel features extracted by CNN in the image domain into a graph representation preserving the vessel topology. Then by exploiting a graph convolutional network (GCN), we enable our model to learn both CNN features and vessel topological features simultaneously. The final predication is attained by fusing the CNN and GCN outputs. Using a publicly available AV-DRIVE dataset and an in-house dataset, we verify the high performance of our VTG-Net for retinal A/V classification over state-of-the-art methods (with ~2% improvement in accuracy on the AV-DRIVE dataset).

## 1. Introduction

Being the only vascular network of the human body that is visible to non-invasive imaging techniques, analysis of retinal vascular structures is a common way to diagnose a number of diseases. Conditions such as arteriovenous nicking, arteriolar constriction, vessel dilation, and tortuosity alteration are vital for examining various cardiovascular diseases, diabetic retinopathy, and hypertension (1–3). Specifically, the arteriolar-to-venular ratio (AVR) gives a key biomarker, critical for quantifying the severity of such diseases. Hence, accurate classification of retinal vessels into arteries/veins (A/V) is of significant clinical interest.

Significant research has been done on automatic A/V classification. Early studies (4) focused on designing hand-crafted features for automatic A/V classification. To exploit the tree-shaped retinal vasculature (5), graph based methods were proposed (3, 6, 7). Such methods used the segmented vessel structures to generate a graph, preserving the vessel topology; the graph was then traversed for accurate vessel classification. Recently, convolutional neural network (CNN) based approaches for A/V classification garnered large interest. In (8), a U-Net (9) based method was used for A/V classification. A SegNet (10) inspired encoder-decoder architecture (11) was proposed for pixel-wise classification. A multi-task framework with spatial activation was given (12) for simultaneous vessel segmentation and classification. Although outperforming traditional graph based methods, CNN approaches still suffer several drawbacks: (i) limited vessel connectivity; (ii) multiple class assignment of a single vessel segment. Recently, Chen et al. (13) proposed a generative adversarial network based method in which a topology preserving module with triplet loss was introduced to address the issue of limited connectivity of classified vessels. But, effective solutions for both these drawbacks of known CNN based approaches still remain highly sought.

**Figure 1:**
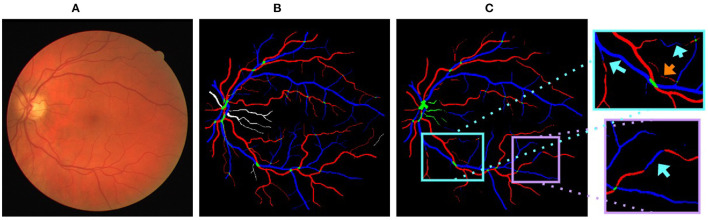
**(A)** A sample fundus image. **(B)** Ground truth of **(A)** arteries, veins, vessel crossings, and uncertain vessels are marked in red, blue, green, and white, respectively. **(C)** Output generated by U-Net (9) on **(A)** (training performed by merging uncertain vessels and vessel crossings into a single class). Cyan arrows highlight vessels with multiple class assignments and orange arrows show limited vessel connectivity.

CNN based approaches commonly use a series of feature extractors (also called spatial filters, kernels, or channels) to extract hierarchical information. Each filter extracts information from a fixed size spatial input neighborhood [the receptive field (14)] and propagates it to the output. Current spatial feature extraction methods are not able to handle the issues of multiple class assignment and limited vessel connectivity well (e.g., see Figure 1). Some seemingly simple cases for the graph based methods (3, 6, 7) can be wrongly classified by CNNs, possibly because their feature extractors do not capture vessel topology effectively. Thus, we believe that incorporating a deep graph-based model that can effectively capture vessel topology into a CNN based approach will improve A/V classification.

Recently, graph convolutional network (GCN) models have been shown to be effective for analyzing graph-structured data. Information propagation on graphs can be formulated, by conditioning the learning models both on such data and the adjacency matrices of the underlying graphs. Known approaches (15–19) have explored graph convolution for learning graph data in various applications, such as e-commerce (customer-product interaction), chemistry (molecule interaction), and citation networks (author-paper interaction). For retinal vessel classification, graph convolution was first proposed in (20) by generating a graph representation with graph nodes defined using sampled skeleton of vessels; graph edge information was extracted from the vessel skeleton, and graph node features were sampled from CNN feature maps using node locations. In (21), a model was proposed by using only vessel pixels as graph nodes, ignoring all non-vessel pixels; graph edges were built using a local patch based neighborhood, and node features were extracted from CNN feature maps using vessel segmentation masks. Although quite effective, these approaches failed to exploit the potential of GCNs by ignoring non-vessel pixels in graph generation and representation.

To improve A/V classification on fundus images by incorporating vessel topological features with CNN features, we propose VTG-Net (vessel topology graph network). VTG-Net exploits graph convolution based learning by strategically transforming the hierarchical CNN features of an input fundus image into a graph representation that preserves vessel topology. Specifically, using a CNN model trained on the input dataset, we first extract image features along with the segmented vessels in the input images. Next, by using CNN features and the segmented vessels (providing the underlying graph structure), a graph representation is produced while preserving the non-vessel pixels as isolated graph nodes. Employing a GCN, we classify the generated graph by extracting its topological features. Lastly, by fusing the CNN output and GCN output, the final prediction is attained.

In contrast to the known GCN based methods for A/V classification, our VTG-Net seeks to address the issue of broken vessels by retaining non-vessel pixels as (isolated) graph nodes. Our approach is hinged on our observation that, if discarding the information content of non-vessel pixels, the errors generated by CNN (disconnected vessels generated due to, e.g., low image quality, lesser model ability) will propagate and cannot be corrected. The inclusion of isolated background nodes may facilitate CNN error correction since GCNs in general leverage not only edge information but also node features for classification. Further, GCN features learned by VTG-net from the connected graph portions (positive vessel examples) can help classify the disconnected portions. Disconnected vessels can still be classified with good accuracy using node features, since graph edges need not necessarily encode node similarity (the same label) (15), which is useful for A/V classification.

We evaluate our VTG-Net using a public dataset AV-DRIVE (22) and an in-house dataset, and our experimental results show its high efficacy.

The rest of this paper is organized as follows. In section 2, our proposed framework is presented. Experimental results are discussed in section 3. Ablation analysis is provided in section 4. Section 5 concludes the paper.

## 2. Method

**Figure 2:**
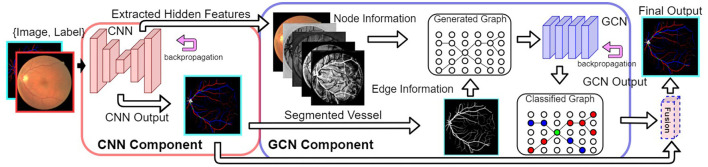
Our proposed VTG-Net framework. Using the CNN component (in the red box), node and edge information for generating our graph representation is extracted. A GCN model is trained using the generated graphs (in the blue box) for topological feature extraction based on the CNN features. The final output is generated by fusing the CNN output and the GCN output.

Figure 2 shows our VTG-Net framework for A/V classification. It consists of three main steps. (1) A CNN (in the red box of Figure 2) is trained using the input dataset. (2) The extracted features and segmented vessels from CNN are used to generate a vessel topology graph. (3) A GCN model is trained using the generated graphs to produce classified output (in the blue box of Figure 2). The final prediction is attained by fusing the CNN output and GCN output.

### 2.1. Graph Convolution Based Topology Analysis

In contrast to standard convolution where information is exchanged only in a small neighborhood (determined by the filter receptive field), graph convolution enables long range information exchange by incorporating adjacency matrices of graphs into message passing (23). Assume an undirected graph **G** = (**V, E**), with *N* nodes *v*_*i*_ ∈ **V** (each node containing *C* features) and *M* edges (*v*_*i*_, *v*_*j*_) ∈ **E**. The edge connectivity (capturing topological neighboring relations) is represented by an adjacency matrix **A** ∈ ℝ^*N*×*N*^. The spectral graph convolution of a tensor *x* ∈ ℝ^*N*×*C*^ with a filter *g*_θ_ is defined as $g_{\theta }⋆x=\text{U}g_{\theta }{\text{U}}^{\text{T}}x$, where **U** is the matrix of eigenvectors of the graph Laplacian matrix **L** (15), which is a matrix representation of the graph **G**. **L** is defined as $\text{L}={\text{I}}_{\text{N}}-{\text{D}}^{-\frac{1}{2}}\text{A}{\text{D}}^{-\frac{1}{2}}$, where **D** (${\text{D}}_{ii}=\sum_{j}\left({\text{A}}_{i,j}\right)$) is a diagonal matrix of node degrees and **I**_**N**_ is the identity matrix (24, 25). To reduce the cost for computing $\text{U}g_{\theta }{\text{U}}^{\text{T}}$, the above graph convolution is approximated using a truncated expression of Chebyshev polynomials *T*_*k*_(*x*) up to the *K*^*th*^ order, i.e., $g_{\theta^{\prime }}⋆x\approx \overset{K}{\underset{k=0}{\sum }}\theta_{k}^{\prime }T_{k}\left(\hat{\text{L}}\right)x$, where $T_{k}\left(\hat{\text{L}}\right)=2\hat{\text{L}}T_{k-1}\left(\hat{\text{L}}\right)-T_{k-2}\left(\hat{\text{L}}\right)$, with $T_{0}\left(\hat{\text{L}}\right)=1$ and $T_{1}\left(\hat{\text{L}}\right)=\hat{\text{L}}$ (26), and $\theta_{k}^{\prime }$ are the filter parameters acting as node feature transformers. The rescaled graph Laplacian matrix $\hat{\text{L}}=\frac{2}{\lambda_{max}}\text{L}-{\text{I}}_{\text{N}}$ (λ_*max*_ is the largest eigenvalue of **L**) can be viewed as an encoder of the topological information of the graph **G**.

In (15), a first order approximation of the Chebyshev polynomial (*K* = 1) is shown to be effective. Using *K* = 1 and λ_*max*_ = 2, the graph convolution can be approximated as:


(1) $$\begin{array}{l} g_{\theta^{\prime }}⋆x\approx \theta_{0}^{\prime }x+\theta_{1}^{\prime }(\text{L}-{\text{I}}_{\text{N}})x=\theta_{0}^{\prime }x-\theta_{1}^{\prime }{\text{D}}^{-\frac{1}{2}}\text{A}{\text{D}}^{-\frac{1}{2}}x\\ \;\approx \theta ({\text{I}}_{\text{N}}+{\text{D}}^{-\frac{1}{2}}\text{A}{\text{D}}^{-\frac{1}{2}})x \end{array}$$

where θ is chosen as $\theta =\theta_{0}^{\prime }=-\theta_{1}^{\prime }$ for constraining the number of parameters. To include self-connections of nodes in localized aggregation ($\hat{\text{A}}=A+I_{N}$) and to avoid vanishing/exploding gradients ($\hat{{\text{D}}_{ii}}=\underset{j}{\sum }{\hat{\text{A}}}_{ij}$), a normalization trick was proposed (15): ${\text{I}}_{\text{N}}+{\text{D}}^{-\frac{1}{2}}\text{A}{\text{D}}^{-\frac{1}{2}}\to {\hat{\text{D}}}^{-\frac{1}{2}}\hat{\text{A}}{\hat{\text{D}}}^{-\frac{1}{2}}$. Applying this normalization trick to Equation (1), the graph convolution can be generalized as:


(2) $$\begin{array}{l} Y={\hat{\text{D}}}^{-\frac{1}{2}}\hat{\text{A}}{\hat{\text{D}}}^{-\frac{1}{2}}X\Theta \end{array}$$

where *X* ∈ ℝ^*N*×*C*^ is the node feature vectors of the graph (*N* nodes with *C* dimensional features), and Θ ∈ ℝ^*C*×*F*^ is the matrix of filter parameters extracting *F* hidden features. *Y* ∈ ℝ^*N*×*F*^ is the output of the graph convolution operation.

Using the graph convolution shown in Equation (2), a neural network model *f*(*X*, **A**) is trained [unlike a standard convolutional model *f*(*X*)) by conditioning *f*(·) simultaneously on the matrix of node features and the adjacency matrix of the graph [${\hat{\text{D}}}^{-\frac{1}{2}}\hat{\text{A}}{\hat{\text{D}}}^{-\frac{1}{2}}X$ in Equation (2]. Further, similar to CNN, by stacking multiple layers performing graph convolution, hierarchical topological features can be extracted by a GCN model. Both the node definition (node features) and graph structure (edge connectivity) play a key role in determining information propagation in GCN. In the next section, we describe how we utilize the extracted CNN features and segmented vessel structures to generate the needed graph representation for our VTG-Net.

### 2.2. Graph Representation Generation

**Figure 3:**
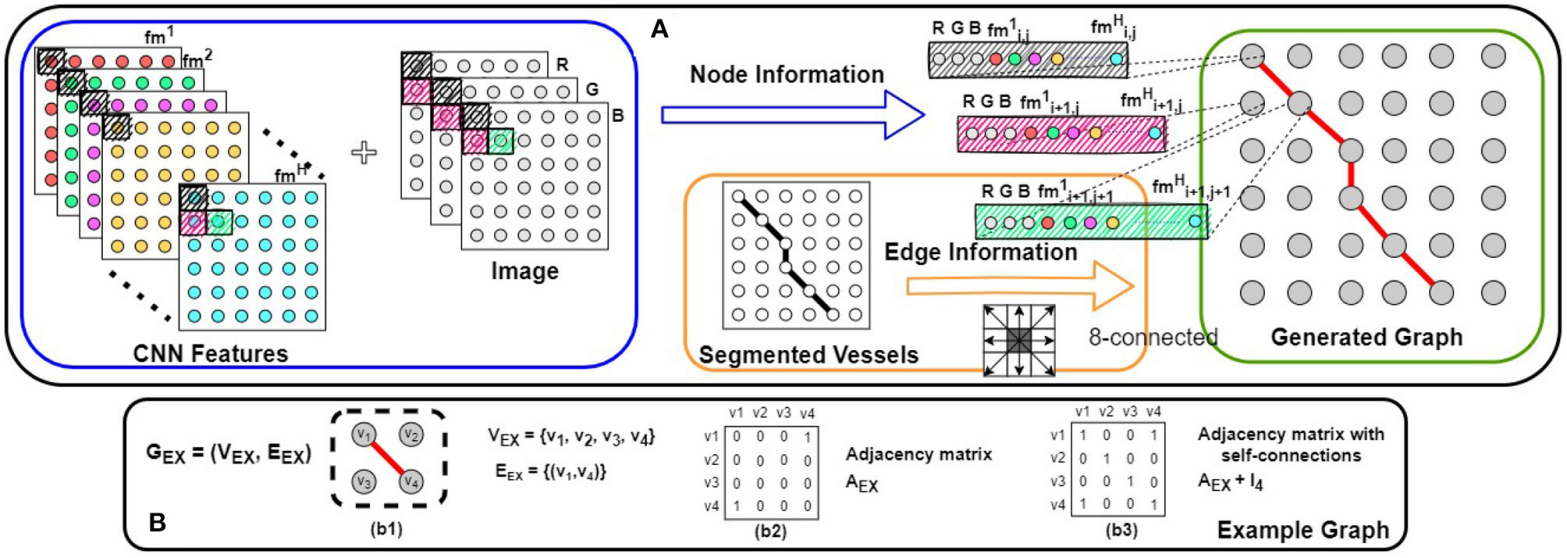
**(A)** Our proposed method for graph representation generation. Combining the input image with the CNN features (shown in the blue box), node features are assigned to the graph. Using the segmented vessel structure, the edge connectivity is determined (shown in the orange box). Combining these two types of information, our graph representation is generated (shown in the green box). In **(B)**, a simple example of an undirected graph [**G**_**EX**_ = (**V**_**EX**_, **E**_**EX**_)] is shown; (b2,b3) the adjacency matrix (**A**) and the adjacency matrix with self-connections ($\hat{\text{A}}$) of this graph.

To leverage a GCN model to incorporate vessel topological features with the extracted CNN features, a graph representation of the CNN features is used. We propose a graph representation, **G** = (**V**, **E**) (*v*_*i*_ ∈ **V**, (*v*_*i*_, *v*_*j*_) ∈ **E**), which can be generated utilizing the CNN features for its nodes and the underlying vessel structure for its edge connectivity. Our proposed method for graph representation generation is illustrated in Figure 3 along with its major components. We first explain the CNN feature extraction, followed by the vessel structure generation. Finally, we combine these two types of information to generate our graph representation.

#### 2.2.1. CNN Feature Extraction

For a CNN performing pixel-wise A/V classification [CNN output $\in {\mathbb{R}}^{P\times Q\times CL_{out}}$, *CL*_*out*_= (background, artery, crossing+unknown, vein)] on an input image $\in {\mathbb{R}}^{P\times Q\times CL_{in}}$ (i.e., height × width × channels), we can assume that the last layer (uppermost) of the network is the classifier, while the remaining layers function as the feature extractor. Utilizing the *H* output features of the feature extractor, the classifier generates the final class probabilities (${\mathbb{R}}^{P\times Q\times H}\to {\mathbb{R}}^{P\times Q\times CL_{out}}$). For instance, a convolutional layer with a 1 × 1 filter $f_{1\times 1}\in {\mathbb{R}}^{1\times 1\times CL_{out}}$ is used as the CNN classifier in (12, 13). Thus, we use the input feature maps of the last 1 × 1 convolution layer as our representative CNN features.

#### 2.2.2. Vessel Structure Extraction

**Figure 4:**
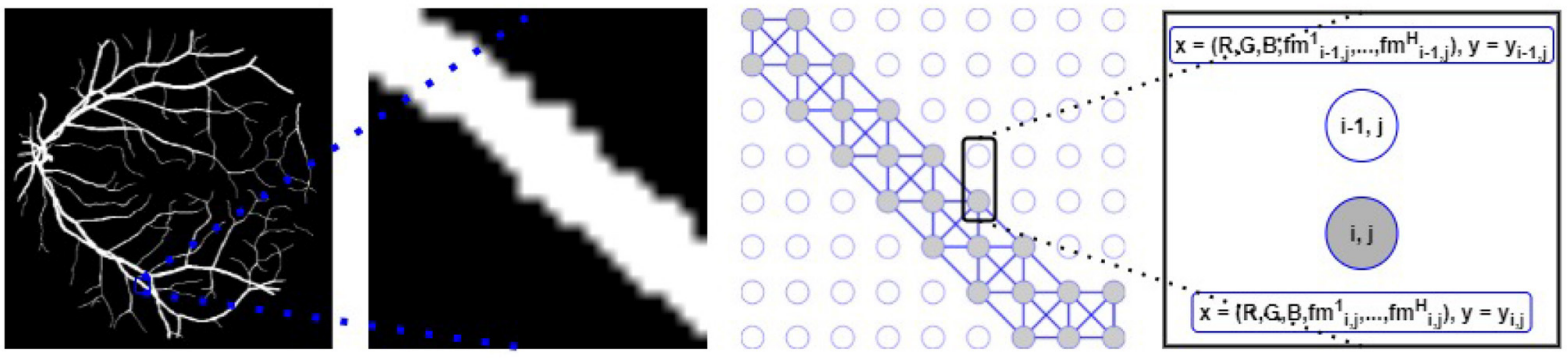
**Left**: Segmented vessels. **Middle-left**: A zoomed-in segmented vessel segment. **Middle-right**: Graph representation with vessel (gray circles) and background (white circles) nodes; graph edges are built following the segmented vessel segment. **Right**: Each node (for both connected vessel nodes and isolated background nodes) is associated with the *x* features and is mapped to the *y* label.

The underlying retinal vessel structure (captured by the segmented vessels) provides a guide on the connectivity among pixels of the input image. Thus, we use the segmented vessels to construct our graph representation. Specifically, each pixel of the segmented vessel mask is treated as a node in **G**. If two adjacent pixels are both classified as the vessel class, an edge connects them in **G**. To identify all vessel pixels, the multi-class pixel-wise classified CNN output is converted to foreground/background classification (i.e., ${\mathbb{R}}^{CL_{out}}\to {\mathbb{R}}^{0,1}$). Then for each node (i.e., each pixel) of our graph representation **G** (*N* = *P* × *Q*), we explore the pixel's 8-connected neighborhood (shown in Figure 3). If and only if both adjacent *v*_*i*_ and *v*_*j*_ belong to the segmented foreground, (*v*_*i*_, *v*_*j*_) ∈ **E** and the adjacency matrix **A** of **G** is updated accordingly. Background pixels (non-vessel pixels) are represented as isolated nodes in **G** (shown in Figure 4).

#### 2.2.3. Graph Representation Generation

Using the map of extracted CNN features and the vessel structure, we generate our graph representation **G** = (**V**, **E**), with *N* nodes and an adjacency matrix **A**. By combining the image channels (*CH*; RGB for fundus images) as additional features (shown in a blue box in Figure 3), each node has a feature vector of length *H* + *CH*. Let *X* ∈ ℝ^*N*×(*H*+*CH*)^ be the set of feature vectors of all the *N* nodes in **G**. Combining *X* and **A**, we are now ready to learn the GCN model *f*(*X*, **A**) [i.e., to determine the values of the parameters of the model *f*(*X*, **A**)] by using Equation (2) for information propagation on our graph representation **G**.

### 2.3. Graph Classification and Fusion

**Figure 5:**
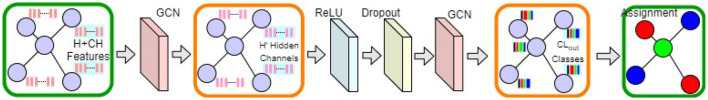
Our proposed GCN model. Input and output graph representations are shown in the green boxes. Intermediate graph activations are highlighted in the orange boxes.

To extract hierarchical (topological) features, we propose a multi-layer GCN model. Our proposed GCN model is shown in Figure 5. Using the graph convolution operation [defined in Equation (2)], *X* is transformed into *H*′ hidden feature channels (ℝ^*N*×(*H*+*CH*)^ → ℝ^*N*×*H*′^; see the left orange box in Figure 5). After activation and dropout (27), another graph convolution operation converts the hidden features into output class probabilities, i.e., ${\mathbb{R}}^{N\times H^{\prime }}\to {\mathbb{R}}^{N\times CL_{out}}$. Using an appropriate loss function (e.g., cross-entropy) and gradient back-propagation, the model parameters (Θ) of the GCN model *f*(*X*, **A**) are learned.

To obtain more accurate classification, we further fuse the pixel-wise classified CNN output (${\mathbb{R}}^{P\times Q\times CL_{out}}$) and the GCN output (${\mathbb{R}}^{N\times CL_{out}}$) to generate the final output of our model (i.e., ${\mathbb{R}}^{P\times Q\times CL_{out}}⊙{\mathbb{R}}^{N\times CL_{out}}\to {\mathbb{R}}^{N\times CL_{out}}$, where ⊙ denotes a fusion operation). One possible way to perform this fusion is to use an agreement based voting scheme in which only an agreement between both these outputs permits a class assignment (e.g., ${\mathbb{R}}_{Fused}^{N\times CL_{i}}={\mathbb{R}}_{CNN}^{P\times Q\times CL_{i}}\cap {\mathbb{R}}_{GCN}^{N\times CL_{i}}$, where *i* = output class). Disagreements between the CNN and GCN outputs are ignored. Another way is to assign weights to the CNN and GCN output class probabilities (${\mathbb{R}}_{Fused}^{N\times CL_{i}}=w_{CNN}{\mathbb{R}}_{CNN}^{P\times Q\times CL_{i}}+w_{GCN}{\mathbb{R}}_{GCN}^{N\times CL_{i}}$, where *w*_*CNN*_ and *w*_*GCN*_ are the weights for the CNN and GCN output class probabilities, respectively). After the fusion of individual class probabilities, a 50% threshold is applied to generate the final output for each class. Various fusion options with experimental details are shown in section 4.5.

## 3. Experimental Evaluation

### 3.1. Datasets

We use a public dataset AV-DRIVE (22) and an in-house dataset (which we call the Tongren dataset) to evaluate our VTG-Net for retinal A/V classification. In the AV-DRIVE dataset (22), 20 training images and 20 test images are provided along with pixel-wise annotation of artery, vein, crossing regions, and uncertain vessels. We merge the crossing regions and uncertain vessels into a single uncertain class for our experiments [$CL_{out}^{AV-DRIVE}=$ (background, artery, crossing+unknown, vein)]. Our in-house dataset contains 30 fundus images collected by the Department of Ophthalmology, Beijing Tongren Hospital, and pixel-wise ground truth annotations were generated by experts for artery, vein, and uncertain vessels [$CL_{out}^{Tongren}=$ (background, artery, unknown, vein)]. Twenty images of the Tongren dataset are used for training and the remaining 10 images are for testing.

### 3.2. Experimental Setup

For the CNN training, we use PyTorch with the *He* initialization (28). To limit overfitting, data augmentation is performed using random flipping and rotation (14). Using a standard U-Net (9) as the CNN model, training is performed. The CNN training uses a cross-entropy loss and the Adam optimizer (29) (β_1_ = 0.9, β_2_ = 0.999, ϵ = 1*e* − 08) with an initial learning rate 2*e* − 05, which is halved in every 10*k* epochs for 20*k* epochs. For graph generation, we stack the input image along with the extracted CNN features (64 feature maps) to generate the node feature vectors [*H* + *CH* = 64 + 3 (RGB) = 67]. After experimenting with different values (128, 64, 32, 16) for the hyperparameter *H*′ (GCN hidden features), 32 is selected. For the GCN training, we use the PyTorch-Geometric framework. The GCN training uses the Adam optimizer (29) (β_1_ = 0.9, β_2_ = 0.999, ϵ = 1*e* − 08) with an initial learning rate 0.003, which is ${\frac{1}{100}}^{th}$ in every 30 epochs for 200 epochs for the AV-DRIVE dataset. For the Tongren dataset, GCN training is performed with an initial learning rate 0.04, which is ${\frac{1}{10}}^{th}$ in every 15 epochs for 200 epochs. Following known approaches, evaluation is performed by treating artery as the positive class and vein as the negative class.

Following known studies (13), we used accuracy, sensitivity, and specificity as evaluation metrics. For variability analysis, experiments are repeated for five times. Mean and standard deviations are used to report the outcome of the experiments. For ablation analysis, only the mean values of the base experiments are used for comparison. Using 2-tailed, paired sample *t*-tests, *p*-values were computed. For *p* < 0.05, observations are considered as statistically significant.

### 3.3. Results

**Figure 6:**
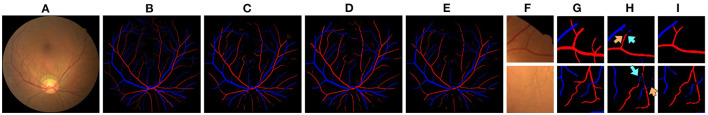
**(A,F)** An image example and two image regions in it. **(B,G)** The corresponding ground truth. **(C–E)** The outputs of CNN, GCN, and VTG-Net. **(H)** CNN-only outputs by our CNN (cyan arrows highlight some vessels with multiple class assignments, and orange arrows mark limited vessel connectivity in the CNN-only outputs, which are rectified by our GCN as shown in **I**).

**Figure 7:**
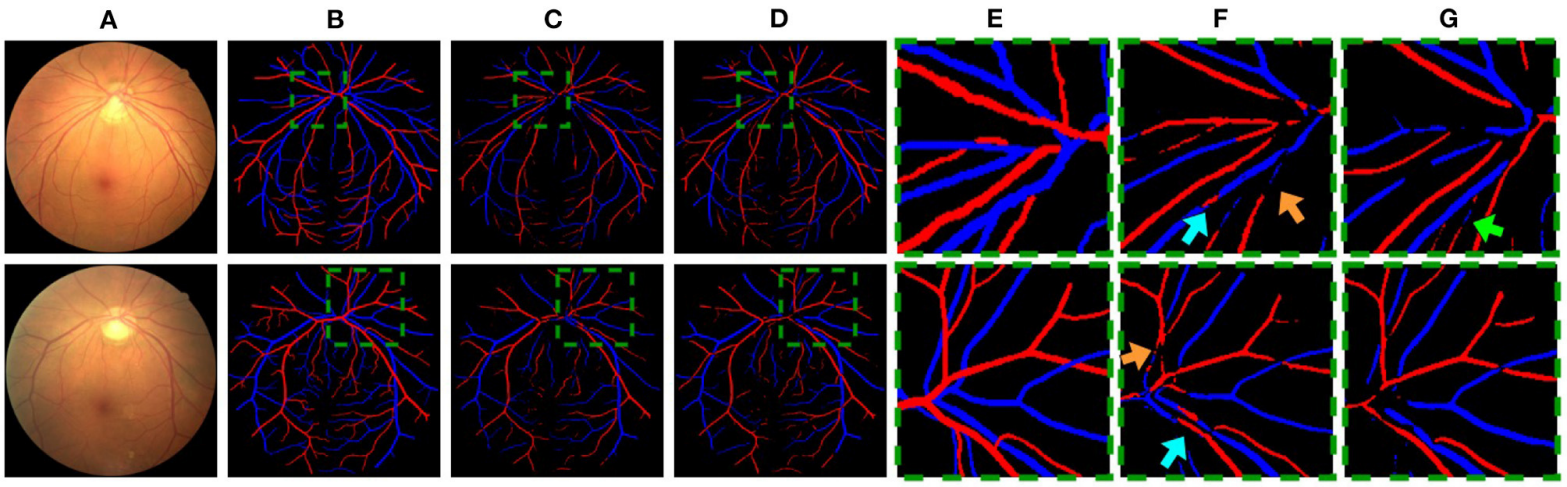
**(A)** Example images. **(B,E)** The corresponding ground truth and zoomed in regions. **(C,F)** CNN outputs. **(D,G)** GCN outputs. In **(F)**, cyan arrows highlight some vessels with multiple class assignments, and orange arrows mark limited vessel connectivity in the CNN-only outputs. In **(G)**, a green arrow highlights a failure case where multiple class assignments are present in the GCN output.

Quantitative results obtained on the AV-DRIVE dataset (22) and the Tongren dataset are shown in Table 1. On the AV-DRIVE dataset, comparison is performed with graph based (3, 6, 7, 30), deep learning (DL) based (8, 12, 13, 31) and GCN based (21) methods. Evaluation under the same criteria as used by known studies reveals that VTG-Net achieves an mean accuracy (Acc) of 98.11% on the AV-DRIVE dataset, outperforming all state-of-the-art methods. In comparison with CNN-only approaches, utilizing GCN improves classification accuracy and sensitivity (with *p* = 0.003 and *p* = 0.004, respectively). The improvement shown in specificity by GCN is found to be not statistically significant (*p* > 0.05) over the CNN-only approaches. Qualitative results of several example cases are given in Figure 6. Improved connectivity and reduction in multiple class assignments achieved by our VTG-Net are highlighted in Figure 6I, compared to the CNN-only outputs shown in Figure 6H. Additional qualitative examples are shown in Figure 7, highlighting the CNN-only outputs and GCN-only outputs. In Figure 7G, a failure case produced by GCN with multiple class assignments is shown. On the Tongren dataset, VTG-Net yields considerably improved classification accuracy compared to the CNN-only method (*p* < 0.05).

**Table 1:** A/V classification results on the AV-DRIVE and Tongren datasets.

		**AV-DRIVE dataset** **(****22****)**		
**Method**	**Type**	**Acc (%)**	**Sen (%)**	**Spe (%)**
(6)	Graph-based	87.4	90.0	84.0
Our	CNN	94.60 ± 0.70	92.51 ± 1.33	96.12 ± 0.52
	GCN	96.49 ± 0.02	96.18 ± 0.04	96.76 ± 0.05
	Fusion	**98.11** **±** **0.03**	**97.32** **±** **0.11**	**98.70** **±** **0.06**
Tongren dataset				
Our	CNN	93.81 ± 0.32	94.02 ± 1.34	93.82 ± 1.4
	GCN	96.05 ± 0.01	95.12 ± 0.07	97.20 ± 0.03
	Fusion	**97.98** **±** **0.03**	**98.01** **±** **0.08**	**98.13** **±** **0.10**

We should mention that a main limitation of our framework evaluation is that the sizes of the datasets used are relatively small. For the public AV-DRIVE dataset, we have followed the standard training/test splitting adopted in existing work [e.g., (12, 13)] in the evaluation, and our VTG-Net has outperformed those methods on this dataset. In our future work, we plan to conduct training and validation of VTG-Net on larger datasets for a more thorough evaluation.

## 4. Ablation Study

We perform systematic ablation study on the graph structures, along with different components of our proposed framework. The ablation experiments use the AV-DRIVE dataset to examine the performances.

### 4.1. Graph Node Choices

Our framework utilizes isolated background pixels along with vessel pixels as graph nodes. In order to analyze the contribution of such isolated nodes, we remove all of them from the graph representation. Experiments are conducted using the graph containing only vessel pixels as graph nodes, and the results are shown in Table 2. It is interesting to see that, in the absence of such isolated nodes, accuracy degrades. In the presence of additional node information, VTG-Net is able to improve classification accuracy.

**Table 2:** Isolated background node contribution using the AV-DRIVE dataset (✓ = with; ✗ = without).

**Method**		**A/V classification**		
**GCN**	**Isolated nodes**	**Acc (%)**	**Sen (%)**	**Spe (%)**
Proposed	✓	**96.49**	**96.18**	**96.76**
	✗	94.66	93.29	95.74

### 4.2. Graph Node Feature Assignment

**Figure 8:**
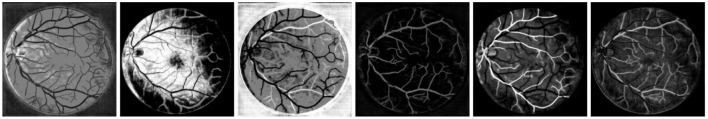
Examples of CNN feature maps. CNN feature maps are used to generate the node features in the graph representation of VTG-Net.

In VTG-Net, each graph node contains CNN features (some examples of hidden feature maps are shown in Figure 8) along with the input image channels (RGB) as node features (*H* + *CH* = 64 + 3 = 67). In order to assess the effect of different node features, we modify the node feature assignment by generating two test cases. In case 1, we use only the CNN features as the node features (*H* = 64); in case 2, only the RGB image is used as the node features (*CH* = 3). Results obtained on the AV-DRIVE dataset are shown in Table 3. The inclusion of the input image as node features has only a little impact on graph classification. In contrast, CNN features are critical for accurate graph classification as the accuracy degrades significantly in the absence of CNN features.

**Table 3:** Node feature contribution analysis using the AV-DRIVE dataset.

**Method**		**A/V classification**		
**GCN**	**Node features**	**Acc (%)**	**Sen (%)**	**Spe (%)**
Proposed	Proposed (67)	**96.49**	**96.18**	**96.76**
	CNN features (64)	96.48	96.17	96.74
	RGB (3)	09.92	00.00	14.99

### 4.3. Graph Edge Arrangement

**Figure 9:**
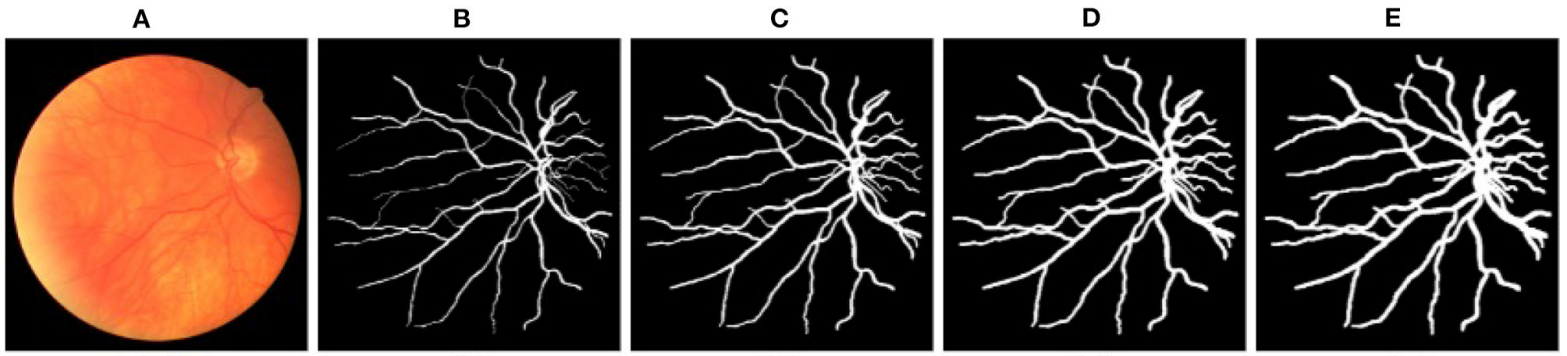
**(A)** A fundus image example. **(B)** The corresponding segmented vessels. **(C–E)** Dilated vessels with a disk-shaped area of radius *r* = 1, 2, 3, respectively.

VTG-Net utilizes CNN-segmented vessels for graph edge assignment (shown in Figure 3), by exploring a node's (pixel's) 8-connected neighborhood. Note that during output generation, CNN may incur some errors in vessel segmentation and classification. As thresholded classification is used to generate the CNN-segmented vessels, errors on segmented vessels may affect the final output generation. Such CNN errors are likely to occur around the segmented vessels (e.g., broken vessels). In order to include the likely error pixels/nodes into the graph structure for possible GCN correction, we explore dilation of the segmented vessels for graph generation. Specifically, during graph generation, we dilate the segmented vessels with different dilation rates to generate the graph representation (e.g., see Figure 9). Using a disk-shaped area of radius *r*, dilation is performed on each segmented vessel pixel (with the pixel as the center of the disk area). Results thus obtained are shown in Table 4. Observe that for a smaller dilation rate (*r* = 1), improvement in accuracy and specificity is observed. But, dilation with a bigger *r* results in accuracy degradation.

**Table 4:** Graph edge arrangement analysis using the AV-DRIVE dataset.

**Method**		**A/V classification**		
**GCN**	**Dilation rate**	**Acc (%)**	**Sen (%)**	**Spe (%)**
Proposed	Segmented (*r* = 0)	96.49	**96.18**	96.76
	*r* = 1	**97.16**	95.49	**98.34**
	*r* = 2	96.07	95.01	96.89
	*r* = 3	95.69	94.39	96.68

### 4.4. Graph Neural Network Models

In VTG-Net, we use GCN (15) to include topological features for A/V classification (as shown in Figure 5). Here, we experiment with various graph message passing techniques to evaluate the effects of different graph convolution models (16, 17, 32). Results obtained are shown in Table 5. Note that the method in (16) (with *k* = 1) exhibits the best specificity. Comparing with GCN (15), other graph convolution models do not show any improvement in accuracy.

**Table 5:** Comparison of different graph neural network (GNN) models using the AV-DRIVE dataset.

	**A/V classification**		
**GNN model**	**Acc (%)**	**Sen (%)**	**Spe (%)**
Proposed [GCN (15)]	**96.49**	**96.18**	96.76
SAGEConv (17)	93.43	90.77	95.51
ChebConv (16) (*k* = 1)	96.26	94.92	**97.22**
ChebConv (16) (*k* = 2)	93.76	92.77	94.58
ChebConv (16) (*k* = 3)	94.16	92.52	95.44
GENConv (32) (*k* = 2)	93.60	91.37	95.32
GENConv (32) (*k* = 3)	93.66	90.81	95.87

### 4.5. Fusion

VTG-Net utilizes an agreement based voting scheme between the CNN output and GCN output to generate the final fusion output (${\mathbb{R}}_{Fused}^{N\times CL_{i}}={\mathbb{R}}_{CNN}^{P\times Q\times CL_{i}}\cap {\mathbb{R}}_{GCN}^{N\times CL_{i}}$, where *i* = output class), as discussed in section 2.3. Here, we experiment with different fusion options by assigning different weights to the CNN and GCN output class probabilities (${\mathbb{R}}_{Fused}^{N\times CL_{i}}=w_{CNN}{\mathbb{R}}_{CNN}^{P\times Q\times CL_{i}}+w_{GCN}{\mathbb{R}}_{GCN}^{N\times CL_{i}}$, where *w*_*CNN*_ and *w*_*GCN*_ are the weights for the CNN and GCN output class probabilities, respectively). To achieve this, each individual class probability is stored separately (without applying *argmax* to both the CNN and GCN outputs), and used for fusion. After fusion, a threshold of 0.5 is used to generate the final output for each class. Results thus obtained are shown in Table 6. Observe that the test cases with higher GCN class probability weights yield better sensitivity and specificity compared to the cases with higher CNN class probability weights. This is expected since the GCN output is a more refined version of the CNN output.

**Table 6:** Comparison of different fusion options using the AV-DRIVE dataset.

	**A/V classification**		
**Fusion**	**Acc (%)**	**Sen (%)**	**Spe (%)**
Proposed	**98.11**	**97.32**	**98.70**
*w*_*CNN*_ = 0.8, *w*_*GCN*_ = 0.2	96.04	94.72	97.10
*w*_*CNN*_ = 0.6, *w*_*GCN*_ = 0.4	96.93	95.74	97.89
*w*_*CNN*_ = 0.5, *w*_*GCN*_ = 0.5	97.18	96.19	97.99
*w*_*CNN*_ = 0.4, *w*_*GCN*_ = 0.6	97.31	96.39	98.07
*w*_*CNN*_ = 0.2, *w*_*GCN*_ = 0.8	97.35	96.43	98.12
*w*_*CNN*_ = 0.1, *w*_*GCN*_ = 0.9	97.23	96.40	97.94

## 5. Conclusions

In this paper, we proposed VTG-Net, a new graph convolution based neural network, for A/V classification. VTG-Net transforms CNN features extracted in an image into a graph representation, preserving vessel topology. Then by exploiting GCN, VTG-Net learns both CNN features and topological features simultaneously. Further, by fusing the CNN output and GCN output, we tackled the two problems of multi-class assignment of a single vessel and limited vessel connectivity. Comprehensive experiments demonstrated the efficacy of our new approach.

## Data Availability Statement

The raw data supporting the conclusions of this article will be made available by the authors, without undue reservation.

## Author Contributions

SM, DC, and XH designed the approach and experiments. SM implemented the method, conducted the experiments, and prepared the manuscript. DC and XH reviewed the manuscript. YW and CW collected the in-house dataset and verified the experimental results. All authors contributed to the article and approved the submitted version.

## Funding

This work was supported in part by the National Science Foundation under Grants CNS-1629914, CCF-1640081, and CCF-1617735, and by the Nanoelectronics Research Corporation, a wholly-owned subsidiary of the Semiconductor Research Corporation, through Extremely Energy Efficient Collective Electronics, an SRC-NRI Nanoelectronics Research Initiative under Research Task ID 2698.004 and 2698.005.

## Conflict of Interest

This study received funding from the Nanoelectronics Research Corporation, a wholly-owned subsidiary of the Semiconductor Research Corporation. The funder was not involved in the study design, collection, analysis, interpretation of data, the writing of this article or the decision to submit it for publication. The authors declare that the research was conducted in the absence of any commercial or financial relationships that could be construed as a potential conflict of interest.

## Publisher's Note

All claims expressed in this article are solely those of the authors and do not necessarily represent those of their affiliated organizations, or those of the publisher, the editors and the reviewers. Any product that may be evaluated in this article, or claim that may be made by its manufacturer, is not guaranteed or endorsed by the publisher.
